# Knowledge and perception of nuclear medicine by radiologists in French-speaking sub-Saharan Africa

**DOI:** 10.22038/AOJNMB.2021.56679.1392

**Published:** 2022

**Authors:** Kokou Adambounou, Koffi Assogba Ahonyi, Gilles David Houndetoungan, Houndetoungan Ouedraogo, Bidamin Ntimon, Fabrice Sodogas, Lantam Sonhaye, Victor Adjenou

**Affiliations:** 1 Biophysic and Medical Imaging departments, Campus Teaching Hospital, University of Lome, Togo; 2 Radiology department, Campus Teaching Hospital, University of Lome, Togo; 3 Department of Biophysics and Nuclear Medicine, Faculty of Health Sciences of Cotonou, University of Abomey- Calavi, Benin; 4 Radiology department, Ouahigouya, University of Ouaga, Burkina Fasso

**Keywords:** Nuclear Medicine, Medical Imaging, Scintigraphic Imaging Radiologists, French-speaking sub-Saharan Africa

## Abstract

**Objective(s):**

to assess the knowledge and perception of nuclear medicine by radiologists in French-speaking sub-Saharan Africa.

**Methods:**

cross-sectional study conducted from April 8 to June 7 2020 including radiologists practicing in French-speaking sub-Saharan African countries. Data were collected electronically via a google form.

**Results:**

Of the 142 radiologists surveyed, 45.8% had already completed an internship in Europe, 3.52% in a nuclear medicine department and 72.54% had a nuclear medicine department in their country of practice. Among these radiologists, 21.13% knew the three main techniques of nuclear medicine and only 9.15% knew that nuclear medicine allows functional, metabolic and molecular studies. On average, 56.8% were aware of clinical indications for the main fields of nuclear medicine. In 47.18% of cases, they thought that scintigraphic imaging was more irradiating than radiological imaging, 71.1% knew about hybrid imaging techniques, 43.66% had read a scientific article on nuclear medicine, 4.93% had attended a nuclear medicine conference and 28.9% had recommended a scintigraphic imaging examination in their report. Half of them would like to see nuclear medicine and radiology merged into a single specialty and 95.77% considered it essential to create a nuclear medicine department in their country.

**Conclusion:**

The level of knowledge of radiologists in French-speaking sub-Saharan Africa about nuclear medicine was, on the whole, unsatisfactory with a generally encouraging perception.

## Introduction

 Nuclear medicine covers all medical uses of radioisotopes (in unsealed sources) for diagnostic or therapeutic purposes (1). The diagnose component of nuclear medicine concerns the diagnosis, prognosis and therapeutic follow-up of a large number of pathologies thanks to two main types of examination: scintigraphy or Single Photon Emission Tomography (SPECT) and Positron Emission Tomography (PET) (2). Its therapeutic component, which concerns the treatment of many cancers, is today in full development, particularly with the personalized therapeutic approach represented by nuclear theranostics (3). Scintigraphic or isotopic imaging is a functional imaging technique that complements morphological imaging techniques (conventional radiology, CT, ultrasound and magnetic resonance imaging). Nuclear medicine is nowadays key in the management of many pathologies. Nuclear medicine and radiology are the two specialties of medical imaging, but unlike radiological imaging which uses X-rays, scintigraphic imaging uses radioactive sources mainly emitting gamma rays (1). Nuclear medicine is developed and accessible in Western countries, the Maghreb and South Africa, unlike in French-speaking sub-Saharan Africa, where very few countries have a nuclear medicine department (4). This lack of technical resources in sub-Saharan African hospitals is partly the reason for medical evacuations to Europe, the Maghreb and South Africa for nuclear medicine examinations (5). 

 In addition to the absence of nuclear medicine departments, nuclear medicine is not also taught in medical schools in the majority of French-speaking sub-Saharan African countries. This situation partly explains the lack of awareness of nuclear medicine in these countries. In 2015, in Togo, for example, in a study evaluating the knowledge and perception of nuclear medicine by Togolese doctors, we noted an unsatisfactory level of knowledge on the subject (6).

 The poor accessibility of nuclear medicine in Africa also raises questions not only about the collaboration between radiologists and nuclear medicine physicians but also about the knowledge and perception of nuclear medicine by radiologists. With this in mind, we undertook this study with the general objective of evaluating the knowledge and perception of nuclear medicine by radiologists in French-speaking sub-Saharan Africa.

## Methods

 This was a descriptive cross-sectional study conducted from April 8 to June 7 2020 and included French-speaking African radiologists practicing in sub-Saharan French-speaking African countries. Sub-Saharan French-speaking African physicians undergoing specialization in radiology (residents), as well as sub-Saharan French-speaking African radiologists not practicing in a sub-Saharan French-speaking African country were not included.

 The parameters studied were the general characteristics of radiologists, their knowledge of medical imaging techniques used in nuclear medicine and their perception and attitude towards nuclear medicine.

 A questionnaire with short open-ended questions (ORQs) and multiple-choice questions (MCQs) designed and saved on Google forms at the address uniform resource locator: https://docs.google.com/forms/d/e/1FAIpQLScZ-6PVdMr9YatNWh6cA8qWFg4LP7YuRWZsdiBWgI6FM2QmJA/viewform was emailed to 173 included radiologists for data collection. These data were recorded in Google-Sheet and then transferred and analyzed in Microsoft Excel 2019 spreadsheet where we made pivot tables with graphs. Statistical tests were carried out using the Chi-2 and Fisher tests according to the variables. Differences were considered statisti cally significant for a p-value of less than 0.05.

## Results

***General characteristics of radiologists***

 The survey forms were completed by 142 radiologists (response rate of 82.1%), 97 of whom were male (68.3%) and 45 female (31.7%), giving a sex ratio of 2.16.

 The most represented countries of origin of the radiologists were Burkina Faso and Togo, and their main countries of practice were Burkina Faso and Côte d'Ivoire; their countries of training were dominated in general medicine by Burkina Faso and Senegal, in radiology and medical imaging by Côte d'Ivoire and Togo (Table 1).

**Table 1:** Distribution of radiologists by country of origin, of practice, of training in general medicine and in radiology

	**Country of origin**		**Current country of practice**			**Country of general medicine**		**Country of specialization**	
	**n**	**%**		**n**	**%**	**n**	**%**	**n**	**%**
Benin	11	7.75		8	5.63	8	5.63	2	1.41
Burkina Faso	41	28.87		39	27.46	36	25.35	30	21.13
Cameroon	10	7.04		9	6.34	8	5.63	10	7.04
Central Africa Republic	3	2.11		2	1.41	3	2.11	0	0.00
Congo	5	3.52		4	2.82	4	2.82	0	0.00
Ivory Coast	10	7.04		22	15.49	9	6.34	38	26.76
Gabon	3	2.11		6	4.23	0	0.0	4	2.82
Guinea	6	4.23		4	2.82	8	5.63	0	0.00
Mali	7	4.93		6	4.23	12	8.45	0	0.00
Morocco	0	0.0		0	0.0	4	2.82	5	3.52
Niger	13	9.15		12	8.45	12	8.45	0	0.00
DRC	5	3.52		4	2.82	6	4.23	1	0.70
Senegal	10	7.04		11	7.75	14	9.86	21	14.79
Chad	4	2.82		1	0.70	2	1.41	0	0.00
Togo	14	9.86		14	9.86	16	11.27	31	21.83
**Total **	**142**	**100**	**142**		**100**	**142**	**100**	**142**	**100**

DRC: Democratic Republic of Congo

 Radiologists with less than 5 years of professional experience were in the majority (86 or 60.56%), 36 radiologists (25.35%) had between 5 and 10 years of professional experience, 12 (8.45%) between 10 and 15 years, and only 8 (6.63%) had been practicing radiology for more than 15 years. 

 Seventy-two (50.50%) radiologists practiced in both the public and private sectors. Thirty-nine (27.46%) practiced only in the public sector, and 31 (21.83%) practiced only in the private sector.

Sixty-five (45.77%) of them had already done an internship in Europe and only five (3.52%) had already done an internship in a nuclear medicine department.

 Of the 142 radiologists who participated in the study, 103 (72.54%) had a nuclear medicine department in their country of practice, including 13 (9.15%) in their hospital of practice and 39 (27.46%) in their city of practice.

***Radiologists' knowledge of nuclear medicine***

 Forty radiologists (28.17%), including a statistically significant majority of those who had previously worked in a nuclear medicine department, knew that X-rays are of the same physical nature as gamma rays; 77 radiologists (47.18%) thought that scintigraphic imaging was more irradiating than radiological imaging and 114 radiologists (80.3%), almost all of whom were university hospitalists, were familiar with the two main specialities of medical imaging, radiology and nuclear medicine (Table 2).

**Table 2:** Distribution of radiologists with good knowledge of the physical nature of X-rays and gamma rays, good knowledge of the radiation level of scintigraphic imaging compared to radiological imaging and good knowledge of the two specialties that make up medical imaging

	**Knowledge of the physical nature of X-rays and gamma rays**			**Knowledge of the radiation level of scintigraphic imaging compared to radiological imaging**			**Knowledge of the specialities that make up medical imaging**		
	**n**	**%**	**p**	**n**	**%**	**p**	**n**	**%**	**p**
**Previous internship in Europe **			0.908			0.653			0.442
Internship completed (n=65)	18	27.69		32	49.23		54	83.08	
No internship (n=77)	22	28.57		35	45.45		60	77.92	
**Previous internship in a NM department**			0.000			0.743			0.259
Internship completed (n=7)	6	85.71		4	57.14		5	71.43	
No internship (n=135)	34	25.19		63	46.67		109	80.74	
**Professional status**			0.896			0.82			0.024
University hospitalists (n=33)	9	27.27		15	45.45		31	93.94	
Hospital practitioner (n=109)	31	28.44		52	47.71		83	76.15	
**Professional experience**			0.274			0.103			0.187
<5 years (n=86)	23	26.74		38	44.19		64	74.42	
5-10 years (n=36)	10	27.78		20	55.56		32	88.89	
10-15 years (n=12)	6	50.00		3	25.00		11	91.67	
>15 years (n=8)	1	12.50		6	75.00		7	87.50	
**Existence of a NM department**			0.442			0.786			0.480
Nowhere (n=39)	11	28.21		16	41.02		34	87.18	
Hospital (n=13)	2	15.38		6	46.15		11	84.62	
City (n=42)	10	28.80		22	52.38		31	73.81	
Country (n=48)	17	35.41		23	47.92		38	79.17	

NM: Nuclear medicine

Table 3 shows that 30 radiologists (21.13%) were familiar with the three main components of nuclear medicine, i.e. scintigraphic imaging, radioimmuno-analysis and Radionuclide therapy, only 13 radiologists (9.15%) were aware that nuclear medicine allows functional, metabolic and molecular studies, and 101 (71.1%) were aware of the possibility of merging scintigraphic and radiological imaging techniques. This table also shows that radiologists' knowledge of the possibility of merging the two types of imaging was statistically significantly related to their professional experience, their professional status and the existence of a nuclear medicine department in their country of practice.

 The average number of radiologists who knew the clinical indications for the main fields of nuclear medicine was 80.7 (56.8%) with extremums of 66 (46.5%) in nuclear neurology and 98 (69%) in nuclear endocrinology.

**Table 3:** Distribution of radiologists with good knowledge of the three main techniques of nuclear medicine, good knowledge of nuclear medicine exploration methods and good knowledge of the fusion of scintigraphic and radiological imaging techniques

	**Knowledge of the 3 main components of nuclear medicine**			**Knowledge of the nuclear medicine exploration methods**			**Knowledge of the fusion of scintigraphic and radiological imaging techniques**		
	**n**	**%**	**p**	**n**	**%**	**p**	**n**	**%**	**p**
**Previous internship in Europe **			0.301			0.536			0.320
Internship completed (n=65)	14	21.54		7	10.77		48	73.84	
No internship (n=77)	16	20.78		6	7.79		53	68.84	
**Previous internship in a NM department**			0.827			0.974			0.550
Internship completed (n=7)	2	28.57		1	14,29		4	57.14	
No internship (n=135)	28	20.74		12	8.89		97	71.85	
**Professional status**			0.113			0.090			0.037
University hospitalists (n=33)	10	30.30		4	12.12		26	78.79	
Hospital practitioner (n=109)	20	18,35		9	8.57		75	68.80	
**Professional experience**			0.112			0.118			0.022
<5 years (n=86)	14	16.28		6	6.98		53	61.63	
5-10 years (n=36)	9	25.00		6	16.67		31	86.11	
10-15 years (n=12)	5	41.67		1	8.33		10	83.33	
>15 years (n=8)	2	25.00		0	0.00		7	87.50	
**Existence of a NM department**			0.000			0.065			0.040
Nowhere (n=39)	15	38.46		0	0.00		30	76.92	
Hospital (n=13)	0	0.00		6	46.15		5	38.46	
City (n=42)	9	21.43		6	14.28		29	69.05	
Country (n=48)	6	12.50		1	2.08		37	77.08	

NM: Nuclear medicine

***Radiologists' perceptions and attitudes towards nuclear medicine***

 One hundred and thirty-six radiologists (95.77%) felt that the creation of a nuclear medicine department was essential for their country, 71 were in favour of merging nuclear medicine and radiology into a single medical imaging specialty and 51 (35.92%) could have chosen nuclear medicine as a medical specialty if the nuclear medicine department existed in their sub-region. The eventual choice of nuclear medicine as a specialty was statistically significantly related to the existence of a nuclear medicine department in the radiologists' country of practice (Table 4).

**Table 4:** Distribution of radiologists who considered it important to establish a nuclear medicine service in their country, of those who could have chosen nuclear medicine as a medical specialty and those in favour of merging radiology and nuclear medicine into a single medical imaging specialty

	**Importance of establishing a nuclear medicine department **			**Possible choice of nuclear medicine as a medical specialty**			**Merger of Radiology and Nuclear Medicine into a single imaging specialty**		
	**n**	**%**	**p**	**n**	**%**	**p**	**n**	**%**	**p**
**Previous internship in Europe **			0.832			0.100			0.238
Internship completed (n=65)	62	95.38		16	24.62		29	44.62	
No internship (n=77)	74	96.10		35	45.46		42	54.55	
**Previous internship in a NM department**			0.633			0.846			0.172
Internship completed (n=7)	5	71.43		2	28.57		4	57.14	
No internship (n=135)	131	97.04		49	36.30		67	49.63	
**Professional status**			0.697			0.443			0.164
University hospitalists (n=33)	32	96.97		10	30.30		13	39.39	
Hospital practitioner (n=109)	104	97.20		41	37.61		58	53.21	
**Professional experience**			0.782			0.249			0.348
<5 years (n=86)	82	95.35		36	41.86		41	47.67	
5-10 years (n=36)	34	94.44		11	30.56		22	61.11	
10-15 years (n=12)	12	100.00		2	16.67		4	33.33	
>15 years (n=8)	8	100.00		2	25.00		4	50.00	
**Existence of a NM department**			0.511			0.023			0.129
Nowhere (n=39)	36	92.31		13	33.33		17	43.59	
Hospital (n=13)	13	100.00		0	0.00		4	30.77	
City (n=42)	40	95.24		16	38.10		20	47.62	
Country (n=48)	47	97.92		22	45.83		30	62.50	

NM: Nuclear medicine

Table 5 shows that 87 radiologists (87.32%) felt it was important for radiologists to spend a semester in a nuclear medicine department during their training, 41 radiologists (28.9%) had already recommended at least once a scintigraphic imaging examination in a radiological report for a better assessment of their patients. This attitude had a statistically significant relationship with the previous completion of a training course in Europe, the status and the professional experience of the radiologists. The table also shows that only 23 radiologists (16.20%) had ever discussed about a patient with a nuclear physician and that this attitude was more common among the university hospitalists with a statistically significant difference.

**Table 5:** Distribution of radiologists who felt that it is important for radiologists to do internship in a nuclear medicine department, of those who have already recommended a nuclear medicine examination in their report and of those who have already discussed a clinical case with a nuclear physician

	**Importance of a nuclear medicine internship for radiologists**			**Previous recommendation of a nuclear medicine examination**			**Previous discussion with a nuclear physician on a clinical case**		
	**n**	**%**	**p**	**n**	**%**	**p**	**n**	**%**	**p**
**Previous internship in Europe **			0.057			0.000			0.361
Internship completed (n=65)	53	42.74		32	49.23		13	20.00	
No internship (n=77)	71	57.26		9	11.69		10	12.99	
**Previous internship in a NM department**			0.386			0.145			0.185
Internship completed (n=7)	5	4.03		3	42.86		2	28.57	
No internship (n=135)	119	95.97		38	28.15		21	15.56	
**Professional status**			0.626			0.027			0.012
University hospitalists (n=33)	28	22.58		15	45.45		13	39.39	
Hospital practitioner (n=109)	96	77.42		26	23.85		10	09.17	
**Professional experience**			0.222			0.019			0.099
<5 years (n=86)	75	60.48		18	20.93		12	13.95	
5-10 years (n=36)	34	27.42		12	33.33		4	11.11	
10-15 years (n=12)	9	7.26		6	50.00		4	33.33	
>15 years (n=8)	6	4.84		5	62.50		3	37.50	
**Existence of a NM department**			0.173			0.873			0.550
Nowhere (n=39)	35	28.23		11	28.21		7	17.95	
Hospital (n=13)	11	8.87		3	23.08		1	7.69	
City (n=42)	33	26.61		14	33.33		9	21.43	
Country (n=48)	45	36.29		13	27.08		6	12.50	

NM: Nuclear medicine

 The teaching of nuclear medicine as an entity in its own right had received a favourable opinion from 103 radiologists (72.54%). Sixty-two radiologists (43.66%) had once read a scientific article on nuclear medicine; while only 7 (4.93%) with a statistically significant difference according to the completion of a previous training course in Europe or in a nuclear medicine department and professional status, had already participated in a nuclear medicine conference (Table 6). 

**Table 6:** Distribution of radiologists who have already read scientific articles in nuclear medicine, of those who have already attended a nuclear medicine conference, and of those in favour of teaching nuclear medicine as a separate entity

	**Previous reading of scientific articles in nuclear medicine**			**Previous participation in a nuclear medicine conference**			**Teaching nuclear medicine as a separate entity**		
	**n**	**%**	**p**	**n**	**%**	**p**	**n**	**%**	**p**
**Previous internship in Europe **			0.238			0.048			0.146
Internship completed (n=65)	32	49.23		6	9.23		51	78.46	
No internship (n=77)	30	38.96		1	1.30		52	67.53	
**Previous internship in a NM department**			0.095			0.000			0.523
Internship completed (n=7)	4	57.14		2	28.57		3	42.86	
No internship (n=135)	58	42.96		5	3.70		100	74.07	
**Professional status**			0.524			0.353			0.677
University hospitalists (n=33)	16	48.48		3	9.09		23	69.70	
Hospital practitioner (n=109)	46	42.20		4	3.67		80	73.39	
**Professional experience**			0.117			0.014			0.597
<5 years (n=86)	32	37.21		1	1.16		64	74.42	
5-10 years (n=36)	17	47.22		3	8.33		24	66.67	
10-15 years (n=12)	7	58.33		1	8.33		8	66.67	
>15 years (n=8)	6	75.00		2	25.00		7	87.50	
**Existence of a NM department**			0.809			0.166			0.168
Nowhere (n=39)	19	48.72		3	7.69		27	69.23	
Hospital (n=13)	6	46.15		2	15.38		12	92.30	
City (n=42)	16	38.10		1	2.38		33	78.57	
Country (n=48)	21	43.75		1	2.08		31	64.58	

NM: Nuclear medicine

## Discussion

 Our study included 142 radiologists from 13 of the 21 French-speaking countries in sub-Saharan Africa, including 100% of the French-speaking countries in West Africa. The difficulty in finding motivated correspondents in southern African countries, for example, to sensitise radiologists to fill in the survey form was one of the reasons why we were unable to reach radiologists in all French-speaking sub-Saharan African countries. The male predominance of our sample with a sex ratio of 2.16 is less than that found by Adigo et al. in 2016 in their study on the frequency and perceived sources of stress among radiologists in French-speaking Black Africa, where the sex ratio was 4 (7). This result can be explained by a progressive increase in the number of female radiologists in French-speaking sub-Saharan Africa over the last five years.

 Only five radiologists (3.52%) had already done an internship in a nuclear medicine department. This low rate can be explained in part by the very poor accessibility of nuclear medicine services in French-speaking Africa. Our results show that 37.46% of radiologists had no nuclear medicine department in their country and only 27.46% had one in their city of practice. This is an illustration of the very low accessibility of nuclear medicine services in our African countries.

 The physical principle of scintigraphy imaging is essentially based on the emission of gamma rays, whereas that of radiological imaging is based on the attenuation or transmission of X-rays. X-rays and gamma rays are both high-energy electromagnetic radiation and are therefore of the same physical nature (8), contrary to what the majority of radiologists surveyed thought (71.83%). The only difference between X-rays and gamma rays is in their origin. X-rays are of electronic origin while gamma rays are of nuclear origin. 

 More than half of the radiologists (52.8%) knew that scintigraphic imaging is not more radiating than radiological imaging. This result is encouraging given that clinical doctors, particularly in Africa, often have a misconception that scintigraphic examinations are very irradiating, more so than all radiological imaging examinations (6). It should therefore be emphasised that the effective dose associated with most routine nuclear medicine examinations is considerably lower than the dose associated with an abdominal CT scan, estimated at 10 mSv (9, 10). For example, the exposure dose from a bone scan is around 4 mSv and that from a thyroid scan is 1 mSv. In the diagnostic investigation of pulmonary embolism, the thoracic CT angiography delivers an average dose of 15 mSv, while the pulmonary perfusion scan delivers only one (1) mSv (9, 11).

 Radiological imaging is part of the medical specialty known as "Radiology" while scintigraphic imaging is part of another medical specialty called "Nuclear Medicine". It is therefore understood that medical imaging brings together two medical specialties, namely radiology and nuclear medicine. It is not limited to radiology, as some doctors, such as about a quarter of the radiologists in our sample, often think. In addition to scintigraphic imaging, nuclear medicine has two other components, namely radionuclide therapy and radioimmuno-assay (2). Unfortunately, the three components of nuclear medicine were known by only 21.13% of the radiologists surveyed. Unlike radiological imaging, which studies the morphology of organs, scintigraphic imaging studies the functional abnormalities of organs, the metabolism of certain tracers (glucose or lipid) and the molecular phenomena of certain pathologies (12). As only 9.15% of radiologists were aware, it is therefore functional, metabolic or molecular imaging, depending on the nature of the radiotracers used.

 Scintigraphic imaging techniques are nowadays often coupled with radiological imaging techniques for better diagnostic performance (13-15). This is referred to as hybrid imaging as is the case with SPECT-CT and PET-CT. It is therefore regrettable to note that about 30% of French-speaking sub-Saharan African radio-logists were unaware of the possibility of merging radiology techniques with those of nuclear medicine. Our work shows that some of the radiologists' good knowledge of nuclear medicine was statistically significantly related to the completion of previous training in Europe in a nuclear medicine department and the existence of a nuclear medicine department in the radiologists' country of practice. These results thus confirm that the unsatisfactory level of knowledge noted in this study is undoubtedly a consequence of the shortage of nuclear medicine services in our African countries.

 Approximately one third of radiologists were unaware of the clinical indications for each of the main fields of nuclear medicine. This result is not encouraging if we know that nuclear medicine, due to a lack of knowledge or preconceived ideas on the part of some doctors, is perceived as a minor or even optional speciality, whereas nuclear medicine diagnostic examinations cover the major part of the field of medicine and contribute to a better patient management, often with a significant clinical impact (1). Thus, FDG-PET imaging for cancer evaluations as well as for the assessments of cerebral function and myocardial viability is a key modality in oncology, neurology, and cardiology, respect-tively (3). Indeed, FDG-PET presents a very high sensitivity in the detection of more than 90% of cancers in staging, re-staging, assessing therapy response and during the follow-up (16). It is capable of revealing additional metastatic foci not detected by standard diagnostic procedures and can also be used to determine the grade of malignancy of a brain tumour and to assess its prognosis (3, 17). 

 In the United States, FDG-PET was performed in more than 2 million patients in 2018, a 7% increase compared with 2017 (18). In addition to FDG, several other tracers like PSMA, Choline, DOTATATE, and Methionine are increasingly used in the management of diverse pathologies such as prostate cancer, central nervous system and neuroendocrine tumors (3, 16, 19). ^68^Ga-PSMA-PET/CT imaging for example, appears clinically to have superseded CT, and appears superior to MR imaging, for the detection of metastatic disease (20). It has the ability to reliably stage prostate cancer at presentation and can select patients who may benefit from targeted systemic radionuclide therapy (20).

 In pulmonology, ventilation/perfusion scintigraphy is one of the validated modalities for the diagnosis of pulmonary embolism, particularly in the case of contraindications to thoracic CT angiography (21, 22). A normal pulmonary perfusion scan eliminates with certainty the diagnosis of pulmonary embolism (2, 21). Unfortunately, a study that we carried out in 2020 noted that the place given to pulmonary scintigraphy by cardiologists in the assessment of pulmonary embolism in sub-Saharan Africa was unsatisfactory (23). In cardiology, myocardial perfusion tomography is a key examination in the early detection and prognostic evaluation of coronary insufficiency (24, 25). In neurology, many pathologies such as brain tumours and neuroblastoma are explored by scintigraphic imaging with good diagnostic performance (21). In paediatrics, solid tumours, osteoarticular infections and malformative uropathies are the most frequent indications for nuclear medicine (26). In urology, dynamic and static renal scintigraphy is a functional, low-radiation and non-invasive means of assessing relative renal function and the patency of the excretory urinary tract (27).

 In view of the above, it is clear that the absence of a nuclear medicine department in several sub-Saharan African countries is an obstacle to better patient care (6). It is therefore gratifying that 95.77% of the radiologists in our study considered the creation of a nuclear medicine service in their respective countries to be essential.

 Our study shows that the perception of nuclear medicine by radiologists is encouraging since not only most of them consider the creation of nuclear medicine services in their countries to be essential, but also about a third of them had stated that they could have chosen nuclear medicine if training in this specialty was available in their sub-regions.

 Nuclear medicine and radiology are two very complementary and very close specialties, and they can be merged into a single medical imaging specialty, as half of the radiologists would like. While the merger of these two specialties into a single medical imaging specialty is already effective in some English speaking countries, this is not the case in France where nuclear medicine has been recognised as an independent specialty since 1988 (European Directive). Thus in France, only the nuclear physician has the required skills to interpret a nuclear medicine examination. Since February 2011, the European Society of Radiology has proposed a merger of nuclear medicine and radiology into an exclusive medical imaging specialty. Nuclear medicine as well as radiology would then become a sub-specialty of this medical imaging specialty with a common core of 3 years of radiology followed by 2 years in nuclear medicine (2). In any case, the training of radiologists in the era of hybrid imaging must include internships in nuclear medicine departments to enable them to become familiar with scintigraphic imaging techniques. It is therefore gratifying to note that 87.32% of the radiologists in our sample felt it was important for radiologists to spend a semester in a nuclear medicine department during their training. The good knowledge of nuclear medicine most often observed among radiologists who had done a nuclear medicine internship with a statistically significant difference in the present study is an illustration of the importance of a nuclear medicine internship in the radiologists' training curriculum. 

 Only 28.9% of radiologists had already recommended a scintigraphic imaging examination in a radiological report for better assessment of their patients, and 16.2% had discussed a patient with a nuclear physician before. This result, which highlights the low level of collaboration between radiologists and nuclear medicine physicians in French-speaking sub-Saharan Africa, can be explained in part by the scarcity of nuclear medicine physicians in sub-Saharan Africa. This low level of collaboration between radiologists and nuclear medicine physicians is also reflected in the low level of interest among radiologists in scientific activities in the field of nuclear medicine, since only 4.93% of the radiologists surveyed had ever participated in a nuclear medicine conference.

 In French-speaking Africa, nuclear medicine, unlike radiology, is not taught in medical schools. This situation is regrettable because the teaching of nuclear medicine as an entity in its own right, as desired by 72.54% of radiologists, or even as a module of the medical imaging teaching unit, would certainly improve the knowledge of future radiologists about nuclear medicine. 

## Conclusion

 The level of knowledge of French-speaking African sub-Saharan radiologists in nuclear medicine was generally unsatisfactory given the proximity of the two specialties, but their perception of nuclear medicine was relatively encouraging. Their attitudes towards nuclear medicine need to be improved in order to promote nuclear medicine in French-speaking sub-Saharan Africa for better imaging exploration of patients.

## Declarations

***Ethics approval and consent to participate***

 An approval from the radiation protection commission of the Togolese Ministry of Health had been obtained. The study did not involve the use of animals. The manuscript has not been submitted to any other journal/site in part or in whole for consideration. It is solely submitted to this journal. The physicians included in this study gave written informed consent to participate in this research.

## Consent for publication

 The physicians included in this study gave written informed consent to publish this research

## Availability of data and material

The data used and/or analyzed during the current study are available from the corresponding author on reasonable request.

## Competing interests

 The authors declare that they have no competing interests.

## Authors' contributions

 K.A. put the idea and the design of the study. KS.A, PA.A and K.A. data collection and have contributed to the conception and design of the manuscript. GD.H, B.N., F.S., L.S. V.A had contributed to the conception and design of the manuscript. All authors have been involved in drafting and revising the manuscript. All authors read and approved the final manuscript.

## Declarations of interest

 None.

## Formatting of funding sources

 This research did not receive any specific grant from funding agencies in the public, commercial, or not-for-profit sectors.
